# Use of AI-methods over MD simulations in the sampling of conformational ensembles in IDPs

**DOI:** 10.3389/fmolb.2025.1542267

**Published:** 2025-04-08

**Authors:** Souradeep Sil, Ishita Datta, Sankar Basu

**Affiliations:** ^1^ Department of Genetics, Osmania University, Hyderabad, India; ^2^ Department of Genetics and Plant Breeding, Banaras Hindu University, Varanasi, India; ^3^ Department of Microbiology, Asutosh College (Affiliated with University of Calcutta), Kolkata, India

**Keywords:** intrinsically disordered proteins, conformational sampling, deep learning, artificial intelligence, MD simulations

## Abstract

Intrinsically Disordered Proteins (IDPs) challenge traditional structure-function paradigms by existing as dynamic ensembles rather than stable tertiary structures. Capturing these ensembles is critical to understanding their biological roles, yet Molecular Dynamics (MD) simulations, though accurate and widely used, are computationally expensive and struggle to sample rare, transient states. Artificial intelligence (AI) offers a transformative alternative, with deep learning (DL) enabling efficient and scalable conformational sampling. They leverage large-scale datasets to learn complex, non-linear, sequence-to-structure relationships, allowing for the modeling of conformational ensembles in IDPs without the constraints of traditional physics-based approaches. Such DL approaches have been shown to outperform MD in generating diverse ensembles with comparable accuracy. Most models rely primarily on simulated data for training and experimental data serves a critical role in validation, aligning the generated conformational ensembles with observable physical and biochemical properties. However, challenges remain, including dependence on data quality, limited interpretability, and scalability for larger proteins. Hybrid approaches combining AI and MD can bridge the gaps by integrating statistical learning with thermodynamic feasibility. Future directions include incorporating physics-based constraints and learning experimental observables into DL frameworks to refine predictions and enhance applicability. AI-driven methods hold significant promise in IDP research, offering novel insights into protein dynamics and therapeutic targeting while overcoming the limitations of traditional MD simulations.

## 1 Introduction

Intrinsically disordered proteins and protein regions (IDPs, IDPRs)^1^ challenge the classical structure-function paradigm of proteins, which posits that a protein’s specific biological function is inherently linked to its unique, stable three-dimensional (3D) structure (Trivedi and Nagarajaram, 2022). This paradigm, deeply rooted in Anfinsen’s thermodynamic hypothesis, has served as a foundational principle of structural biology (Dishman and Volkman, 2018). However, IDPs defy this classical view by existing as highly dynamic ensembles of interconverting conformations rather than adopting a single, stable structural state under physiological conditions (Kulkarni et al., 2022). The intrinsic disorder observed in IDPs is a consequence of their distinctive amino acid compositions. These proteins are typically enriched in polar and charged residues—such as serine, glutamine, and lysine—and are depleted in hydrophobic residues, which are essential for forming the stable hydrophobic cores characteristic of folded proteins (Uversky, 2013). The absence of such hydrophobic cores prevents the stabilization of a defined 3D structure, resulting in an ensemble of flexible, unstructured conformations (Orosz and Ovádi, 2011). This structural plasticity allows IDPs to explore a wide conformational landscape, which, in turn, enables functional versatility and adaptability. The dynamic nature of IDPs is central to their functional repertoire, particularly in cellular processes requiring molecular flexibility and promiscuous interactions (Aftab et al., 2024). IDPs mediate interactions with multiple molecular partners through mechanisms such as conformational selection and induced fit, enabling high specificity despite their structural heterogeneity (Arai et al., 2024). This adaptability is often modulated by post-translational modifications (PTMs), which act as molecular switches that fine-tune their interactions and activity (Bah and Forman-Kay, 2016). IDPs frequently serve as hubs or scaffolds in signal transduction pathways, where they coordinate the assembly and function of multi-protein complexes (Wright and Dyson, 2015). Their structural flexibility facilitates the simultaneous or sequential binding of diverse signaling molecules, ensuring efficient signal propagation and integration (Su et al., 2024). This ability to accommodate multiple partners is critical for forming dynamic, reversible interactions that are responsive to cellular stimuli (Kulkarni et al., 2021). In transcriptional regulation, IDPs play pivotal roles by modulating transcription factors and assembling transcriptional complexes (Tsafou et al., 2018). Their structural flexibility enables interactions with diverse DNA sequences and protein partners, facilitating dynamic responses to developmental and environmental cues (Bugge et al., 2020; Salladini et al., 2020). Disordered regions are frequently enriched in host-pathogen protein-protein interactions (PPIs), providing the necessary structural plasticity for dynamic and promiscuous binding (Aftab et al., 2024). Notably, studies analyzing molecular mimicry in host-microbe interactions report that around 78% of bacterial mimicry proteins and 73% of viral mimicry proteins exhibit moderate to high levels of intrinsic disorder, facilitating their roles in immune evasion and host adaptation. Furthermore, 45% of bacterial and 31% of viral mimitopes (small peptide mimics) also fall into the intrinsically disordered category (Garg et al., 2022).

However, the intrinsic flexibility presents significant challenges for traditional methods of structure determination, particularly in accurately sampling the diverse conformational landscapes of these proteins (Roca-Martinez et al., 2022). Conventional structural biology techniques, such as X-ray crystallography and cryo-electron microscopy, rely on the ability to capture proteins in a single, well-defined conformation to generate high-resolution structural data (Evans et al., 2023). The dynamic and heterogeneous nature of IDPs precludes the formation of the ordered crystals required for X-ray diffraction studies, as their lack of a stable tertiary structure prevents them from adopting the uniform conformations necessary for crystal lattice formation (Smyth and Martin, 2000). Moreover, techniques like nuclear magnetic resonance (NMR) spectroscopy and small-angle X-ray scattering (SAXS), while more suitable for studying dynamic systems, face limitations when applied to IDPs. NMR spectroscopy can provide information on the ensemble-averaged properties of IDPs, but the rapid interconversion between conformations leads to broad and overlapping signals, complicating spectral interpretation and making it difficult to resolve individual conformational states (Maiti et al., 2024). Similarly, SAXS yields low-resolution data that represent an average overall conformation present in solution, which can obscure transient or low-population states that may be functionally relevant (Brosey and Tainer, 2019).

A notable example of MD-based conformational ensemble exploration is the study of ArkA, a proline-rich IDP from yeast actin patch kinase Ark1p, which regulates actin cytoskeleton assembly. Using Gaussian accelerated MD (GaMD) (Wang et al., 2021), researchers captured proline isomerization events, revealing that all five prolines in Ark1p significantly sample the cis conformation. This led to a more compact ensemble with reduced polyproline II (PPII) helix content, aligning better with *in-vitro* circular dichroism (CD) data. Biologically, proline isomerization may act as a switch, regulating ArkA’s binding to the SH3 domain of Actin Binding Protein 1 (Abp1p). Since SH3 domains prefer PPII helices, the cis state may slow or modulate binding, affecting signal transduction and actin dynamics, highlighting a broader IDP regulatory mechanism, where conformational switching influences protein interactions (Alcantara et al., 2021).

Traditional MD simulations, while valuable for exploring protein dynamics, are often insufficient on their own to fully capture the conformational landscapes of IDPs due to practical limitations in sampling efficiency and force field accuracy (Zhu et al., 2024a). Beyond these limitations, the sheer scale of the conformational space accessible to IDPs poses another challenge. As a result, there has been a burgeoning interest in leveraging AI-based methodologies to efficiently sample the conformational space in IDPs (Gupta et al., 2022). The advent of a data-rich era in molecular and structural biology, fueled by the exponential growth of high-throughput experimental techniques and computational simulations (Velankar et al., 2021), has provided unprecedented opportunities for the development of data-driven approaches to tackle longstanding challenges in the study of protein structural dynamics (Mura et al., 2018). In this data-rich landscape, DL approaches have demonstrated significant potential in modeling complex biological systems due to their ability to learn intricate, non-linear relationships from large datasets without explicit programming of physical laws (Patel and Tewari, 2022).

In this review, we summarize recent advancements in the application of DL methods to model the conformational ensembles in IDPs (Erdős and Dosztányi, 2024). By examining various DL architectures employed for the purpose, we highlight their potential in capturing the structural dynamics of IDPs that are crucial for understanding their multifaceted biological functions and roles in diseases (Brotzakis et al., 2023; Ruzmetov et al., 2024). Additionally, we explore the integration of experimental data with computational models, emphasizing how interdisciplinary efforts are enhancing our ability to characterize IDP behavior (Zhang O. et al., 2023; Liu et al., 2024b). Furthermore, we also discuss the challenges faced by these generative models in the context of conformational sampling in IDPs and how incorporating physics-based constraints can help in overcoming the energy landscape in IDPs (Guan et al., 2024; Jing et al., 2024).

## 2 Limitations and latest advents of molecular dynamic simulations in sampling conformational ensembles in IDPs

MD simulations have been a fundamental tool in computational structural biology for decades, allowing researchers to explore the atomic-level motions of proteins and other biomolecules over time. In the context of globular proteins, MD simulations can provide detailed insights into the structural dynamics and conformational changes, often pertaining to their function (Hollingsworth and Dror, 2018). However, when applied to IDPs, MD faces several inherent limitations. One of the primary challenges is the sheer size and complexity of the conformational space that IDPs can explore (Bhattacharya and Lin, 2019). IDPs, by definition, do not adopt a single, well-defined structure; instead, they exist as an ensemble of nonconvertible conformations (Bandyopadhyay and Basu, 2020; Kulkarni et al., 2022). Capturing this diversity requires simulations that span long timescales—often microseconds (μs) to milliseconds (ms) — to adequately sample the full range of possible states. Furthermore, MD simulations often start production runs with different random seeds when assigning initial velocities to atoms, typically using a Maxwell-Boltzmann distribution, to ensure that the results are not biased by specific initial conditions (Roy et al., 2014; Roy et al., 2015). Such simulations are computationally intensive, requiring significant computational resources and time, which limits the practicality of MD for large-scale studies of IDPs (Shrestha et al., 2021). Moreover, even with extensive simulation times, MD may fail to sample rare conformations that are biologically relevant but occur only transiently. These rare states can be crucial for the functional role of IDPs in processes such as protein-protein interactions or the formation of transient complexes (Han et al., 2017; Roy et al., 2022). The inherent bias of MD simulations towards sampling states near the initial conditions further complicates the accurate representation of the full conformational ensemble (Sullivan and Weinzierl, 2020). To overcome these challenges, researchers have developed specialized MD techniques tailored to IDPs. Coarse-grained (CG) models, for instance, reduce the level of detail by grouping atoms into larger moieties, thereby lowering computational costs and enabling the simulation of longer timescales, which are critical for capturing the full range of IDP conformations (Hu et al., 2024). Additionally, enhanced sampling methods, such as replica exchange MD (REMD) and metadynamics, are designed to overcome the sampling bias of traditional MD by facilitating the exploration of the entire energy landscape (Han et al., 2017). These methods are particularly effective in identifying and characterizing rare conformational states that play key roles in the biological functions of IDPs (Gong et al., 2021).

A significant obstacle in MD simulations arises from the lack of a precise energy function to guide these methods, particularly in the context of IDPs. Traditional force fields, which are often optimized for globular proteins, may not adequately capture the unique dynamic properties of IDPs, leading to biased sampling and incomplete exploration of conformational space (Schlick et al., 2021). Traditional force fields, primarily developed and optimized for globular proteins, such as AMBER, CHARMM, GROMOS, and OPLS - all of which have an inherent bias towards well-defined secondary and tertiary structures (Guvench and MacKerell, 2008). To overcome this bias, researchers have developed IDP-specific force fields that are better suited to model the unique dynamic properties of disordered proteins (Mu et al., 2021). These force fields, such as CHARMM36 m (Huang et al., 2017), ff14IDPSFF (Song et al., 2017), a99SB-disp (Robustelli et al., 2018), ESFF1 (Song et al., 2020), among others, have been designed with modified parameters (assigning appropriate weightages to the terms) to better account for the lack of stable secondary structures replaced by the malleable, fluid-like nature of IDPs. For example, IDP-specific force fields may reduce the bias towards forming helices and sheets, allowing the simulations to more accurately reflect the true conformational flexibility of IDPs (Song et al., 2020). AWSEM-IDP and MOFF are some CG force fields that were developed for IDP-specific simulation applications (Wu et al., 2018; Latham and Zhang, 2019). Additionally, the choice of solvent model can be just as important as the choice of force field (Fischer et al., 2024). Explicit solvent models like TIP4P/2005 or SPC/E are often preferred because they provide a more accurate representation of water’s dielectric properties and hydrogen-bonding capabilities, which are essential for capturing the highly dynamic and flexible nature of IDPs (Mu et al., 2021). Implicit solvent models, like ABSINTH (Choi and Pappu, 2019), use additional potentials rather than simulated models of water molecules to describe the influence of solvent (Mu et al., 2021; Janson et al., 2023). Addressing post-translational modifications (PTMs) are crucial in conformational sampling of IDPs because they can induce localized changes in charge distribution, hydrophobicity, and steric hindrance, which significantly alter the conformational landscape. These modifications can shift the equilibrium between different conformational states, wherein changes in surface properties modulate binding affinities with molecular partners. They can further create or disrupt transient structural motifs, thereby directly influencing the functional dynamics of IDPs in cellular processes. While several force fields, such as AMBER and CHARMM, have incorporated parameters for common PTMs like phosphorylation and glycosylation, these modifications are not yet fully optimized for IDPs (Mu et al., 2021). Also, MD simulations struggle to effectively integrate experimental data, such as distance restraints or chemical shifts from NMR, global structural features from SAXS, and volumetric density constraints from Cryo-EM to bias the conformational sampling towards experimental profiles. Without the ability to dynamically adjust simulation parameters based on real-time data, the generated ensembles may miss critical structural dynamics and functional states, leading to models that do not accurately reflect the biological reality of IDPs (Wang et al., 2019; Vani et al., 2023; Wang T. et al., 2024). As a result, while MD remains a valuable tool for studying specific aspects of IDP dynamics, its limitations underscore the need for alternative approaches like DL that can more effectively and efficiently sample the vast conformational landscapes of IDPs within feasible computational timescales (Yang et al., 2023).

## 3 The emergence of deep learning methods in protein structure prediction

Deep Learning (DL) is a special kind of machine learning (ML) that utilizes artificial neural networks with multiple layers, often referred to as deep neural networks, to autonomously learn hierarchical representations from complex and large-scale datasets. In recent years, DL has emerged as a preferred tool in computational biology, particularly in the field of protein structure prediction (Pakhrin et al., 2021). Unlike traditional methods that rely heavily on physical principles or manual engineering of input feature vectors, DL models can automatically learn complex patterns and representations from large datasets (Ahmed et al., 2023). The success of DL in predicting the structures of well-folded proteins has been exemplified by groundbreaking projects such as AlphaFold (Ruff and Pappu, 2021) and RoseTTA fold (Baek et al., 2021), which demonstrated the potential of these models to achieve near-experimental accuracy in protein structure prediction (Elofsson, 2023). This success has naturally led to interest in applying similar techniques to the more challenging problem of predicting the conformational ensembles of IDPs.

DL models excel in capturing the intricate relationships between amino acid sequences and their corresponding structural features (Kumar and Srivastava, 2024). These models, particularly those based on convolutional neural networks (CNNs), Recurrent Neural Networks (RNNs), and transformers, can process vast amounts of sequence and structural data, learning to predict not just a single static structure but an entire range of possible conformations (Ferruz et al., 2023). Recent work has emphasized the importance of quantifying ensemble diversity in generative models to ensure comprehensive conformational sampling, aligning with the heterogeneous nature of IDP ensembles (Chinnam et al., 2023; Wu et al., 2018; Ortega et al., 2022). CNNs and RNNs laid the initial groundwork for sequence-based predictions by leveraging local sequence motifs (in CNNs) and short-to medium-range dependencies (in RNNs). CNNs apply sliding filters (kernels) across the sequence to detect local motifs, leveraging multiple stacked layers to progressively extract higher-level features (Alzubaidi et al., 2021). RNNs process sequences one element at a time, updating a hidden state that carries information forward; variants like Long Short-Term Memory (LSTM) or Gated Recurrent Units (GRU) are often employed to mitigate vanishing or exploding gradients. However, their capacity to represent long-range interactions and highly flexible structures is limited, making them less optimal for the extensive conformational ensembles characteristic of IDPs (Mienye et al., 2024). Transformers, a type of DL model originally developed for natural language processing, utilize self-attention mechanisms to weigh the relationships between all elements in a sequence simultaneously, making them particularly powerful for capturing complex dependencies across long protein sequences (Vaswani et al., 2017; Chandra et al., 2023). This ability is particularly advantageous for IDPs, whose structural flexibility results in a wide array of potential conformational states. By leveraging large-scale datasets, such as those available from the Protein Data Bank (PDB) or specialized IDP databases like DisProt (Sickmeier et al., 2007), MobiDB (Piovesan et al., 2021), FuzDB (Hatos et al., 2022), and IDEAL (Fukuchi et al., 2012), DL models can be trained to recognize the diverse conformational patterns characteristic of IDPs. The Protein Ensemble Database (PED) is a primary resource deposit for structural ensembles of IDPs used to train DL models (Ghafouri et al., 2024). This data-driven approach allows DL to sample the conformational landscape of IDPs more comprehensively and efficiently than traditional MD simulations, making it a preferred method in modern structural biology research (Zhu et al., 2024a).

## 4 Deep learning models employed in the conformational sampling of IDPs

To effectively sample the conformational ensembles of IDPs, DL models employ a variety of sophisticated techniques designed to model the high-dimensional and complex nature of IDP conformations. These models range from transformer-based architectures like AlphaFold (pipelines using AlphaFold and its extensions) (Brotzakis et al., 2023; Ghafouri et al., 2024) and variants (Chennakesavalu and Rotskoff, 2024), which leverage sequence-structure dependencies, to generative models such as variational autoencoders (VAEs) (Zhu et al., 2023), generative adversarial networks (GANs) (Janson et al., 2023), and diffusion probabilistic models (Janson and Feig, 2024; Zhu et al., 2024b), each uniquely suited to the conformational sampling challenges of IDPs. By utilizing vast data-driven frameworks, these DL approaches enable efficient and comprehensive exploration of IDP conformational space, often functioning independently of, or in conjunction with, traditional MD simulations. Additionally, certain DL models integrate energy-based principles (Patel and Tewari, 2022; Aranganathan et al., 2024), notably through Boltzmann Generators (BGs) (Patel and Tewari, 2022), to navigate free energy landscapes, reflecting the thermodynamic properties inherent to IDPs. While ensemble learning techniques such as boosting, bagging, and stacking have been widely used in various ML domains, their direct application to IDP conformational ensemble generation remains unexplored albeit promising, for example, incorporating multiple models with physics-based learning and structural features. Most existing machine learning models in IDP research focus on disorder prediction rather than leveraging multiple classifiers to enhance conformational ensemble sampling (Eickholt and Cheng, 2013; Jones and Cozzetto, 2015).

### 4.1 Generative adversarial networks

GANs were one of the first DL-based methods to be used to generate the conformational ensemble of IDPs (Erdős and Dosztányi, 2024). GANs employ a generator-discriminator architecture, where the generator synthesizes novel protein conformations by transforming random noise or latent variables into structural representations, while the discriminator evaluates their plausibility by comparing them against real data, such as contact maps or inter-residue distance distributions derived from experiments or simulations (Gui et al., 2020). The adversarial training process ensures iterative refinement, with the generator learning to produce increasingly realistic ensembles and the discriminator improving its ability to distinguish physically plausible conformations (Zheng et al., 2023).

By training on CG and all-atom MD simulations of IDPs with lengths from 20 to 200 residues, idpGAN learns the underlying distribution of protein conformations specific to different sequences. This approach allows idpGAN to generate accurate and diverse ensembles in a fraction of the computational time required by traditional MD simulations. The efficacy of idpGAN is benchmarked against MD-generated ensembles, demonstrating its ability to produce accurate conformational distributions while achieving orders of magnitude faster sampling. Evaluations against reference MD data reveal that idpGAN-generated ensembles accurately reproduce key structural properties, including residue contact maps, radius of gyration (Rg) distributions, and energy landscapes. Moreover, idpGAN exhibits transferability to an extent, generating ensembles for sequences outside its training set highlighting generalizability. One of the notable advantages of idpGAN is its ability to circumvent the kinetic barriers that constrain MD simulations. Because generative models directly learn the equilibrium distribution of conformational states, they are not bound by timescale limitations inherent to physics-based sampling methods. However, while idpGAN effectively models static conformational distributions, it does not retain temporal information or transition pathways between states. Future developments could integrate energy-based constraints to improve structural realism by ensuring that generated conformations align with biophysically valid energy landscapes. Additionally, hybridizing generative adversarial learning with reinforcement learning (RL) techniques, a ML framework where an agent learns optimal decision-making by interacting with an environment and receiving feedback through rewards, could introduce trajectory-conscious sampling, where RL rewards transition pathways that align with experimentally inferred kinetic data (Janson et al., 2023).

### 4.2 Variational autoencoders

Another of the most promising approaches for generating IDP conformational ensembles is the application of Variational Autoencoders (VAEs) (Liu et al., 2023), which offer a robust framework for learning the underlying statistical distribution of protein conformations from training data (Janson et al., 2023; Zhu et al., 2023). Designed as an extension of traditional (generative) autoencoders (AEs), VAEs employ a dual neural network architecture—a combination of an encoder and a decoder—to reduce the high-dimensional input data, such as protein structural coordinates, into a lower-dimensional latent space, which can then be reconstituted into the original structural format (Kingma and Welling, 2022). This latent space encodes a smooth distribution of conformations, such that novel and realistic protein structures can be generated by sampling from it, offering a means to access structural variations that extend beyond the training set (Chien, 2019). Specifically, for IDPs, VAEs have proven invaluable in capturing the flexibility and structural diversity inherent to these proteins, thereby facilitating the exploration of conformational ensembles that include rare or transient states (Zheng et al., 2023; Zhu et al., 2023). VAEs trained on IDP data have shown a remarkable ability to generate high-quality, experimentally-consistent ensembles with fidelity levels that exceed traditional MD and even AlphaFold-based predictions (Mansoor et al., 2024). Here, protein backbone positions were encoded, providing a compressed yet information-dense representation that, upon decoding, could predict high-quality ensemble structures consistent with IDP conformational fluidity.

**FIGURE 1:**
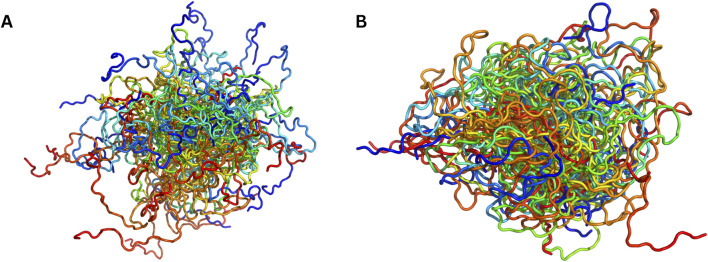
Visual comparison of MD-sampled versus AI-sampled IDP ensembles of α-synuclein (140 residues). **(A)** depicts a superposition of MD-sampled conformations (Allison et al., 2009). **(B)** shows an equivalent number of conformations [sampled by idpGAN (Janson et al., 2023)]. Both the ensembles have been procured from PED and sampled evenly.

The model described in Zhu et al. (2023), employs a VAE framework optimized for IDP ensemble sampling, enhancing the conformational space coverage while maintaining structural consistency with experimental and MD-derived data. The encoder in this model transforms high-dimensional Cartesian coordinate representations of IDP conformations into a low-dimensional latent space using deep neural networks. Unlike AEs, which directly compress data into a deterministic latent space, the VAE applies variational inference, encoding each conformation as a Gaussian-distributed latent variable rather than a fixed vector. To ensure a stable and generalizable latent representation, the model applies Kullback–Leibler (KL) divergence regularization, which enforces the latent space to approximate a Gaussian prior distribution. This prevents overfitting and ensures that interpolations between sampled conformations remain structurally plausible rather than generating unrealistic outliers. Once trained on MD-derived conformations, the decoder samples new latent variables from the learned Gaussian distribution and reconstructs full protein backbone structures. The decoding process follows a hierarchical generative approach, progressively reconstructing structural features from global backbone topology to finer atomic-level details. Unlike traditional AEs, which can suffer from discontinuities in sampled conformations, the VAE framework ensures smooth and diverse ensemble generation, producing conformations that faithfully match experimental chemical shifts, Rg values, and secondary structure distributions. Zhu et al. validated the effectiveness of the generated ensembles by benchmarking them against MD-derived reference structures across five IDP systems (RS1, Aβ40, PaaA2, R17, and α-synuclein). The comparison (e.g., for α-synuclein, Figure 1) demonstrated that the VAE-generated ensembles exhibited significantly lower Cα root-mean-square deviations (RMSD) than those obtained from MD-sampled conformations, indicating improved structural accuracy. Furthermore, Spearman correlation coefficients showed that the generated ensembles better preserved the statistical distribution of MD-derived conformations, reinforcing their consistency with established IDP ensemble characteristics. The model was also experimentally validated using chemical shift predictions and Rg comparisons, demonstrating strong agreement between VAE-generated structures and experimental IDP data (Zhu et al., 2023).

Phanto-IDP (Zhu et al., 2024a) uses an encoder-decoder architecture optimized for IDPs conformational sampling, specifically addressing IDPs’ unique structural flexibility and complexity. The model leverages a graph-based VAE for encoding structural features and a transformer-based decoder for high-fidelity conformation generation. The encoder component of Phanto-IDP employs a graph convolutional network (GCN) to represent protein backbone atomic features as graph nodes and their spatial relationships as edges. This graph-based representation ensures that local and global structural constraints are preserved during encoding. The encoded features are then processed through a variational inference module, where the model learns a probabilistic latent space that allows for continuous conformational sampling. The decoder consists of three transformer blocks, each incorporating self-attention mechanisms and update layers to refine the generated structures. Unlike traditional VAEs, which often struggle with capturing intricate protein folding landscapes, the transformer-based decoder enhances non-local sequence interactions, making it particularly effective for IDP ensemble generation. This approach enables Phanto-IDP to generate ensembles that faithfully reproduce structural properties observed in MD simulations, such as Rg distributions, backbone dihedral angle variations, and contact maps. Phanto-IDP’s performance is benchmarked against both MD-derived ensembles and other generative deep-learning models (Gupta et al., 2022; Zhu et al., 2023). Comparative analysis reveals that Phanto-IDP achieves higher structural fidelity and diversity while significantly reducing computational cost. The reconstructed backbones exhibited an average RMSD of less than 1Å from MD reference conformations (Zhu et al., 2024a).

The Internal Coordinate Net (ICoN) model offers another VAE-based framework for IDP ensemble generation, utilizing bond-angle-torsion (BAT) internal coordinates to represent conformational diversity efficiently. The encoder in ICoN is designed to compress high-dimensional protein conformations into a smooth, three-dimensional latent space, enabling an efficient and compact representation of structural variability. Unlike Cartesian coordinate representations, which may introduce discontinuities in flexible protein structures, ICoN leverages BAT coordinates to capture the essential degrees of freedom governing IDP conformational changes. The encoder architecture consists of multiple fully connected layers, which gradually reduce the dimensionality of the input structural data to a 3D latent space. The model is trained using variational inference. Once trained, ICoN samples new latent vectors from the learned probabilistic distribution, reconstructing full atomistic protein structures via its decoder network. The decoder reverses the encoding process, mapping latent vectors back to BAT coordinates before converting them into Cartesian space. Unlike conventional VAEs, which often struggle to maintain sidechain and backbone coherence, ICoN directly learns the physical constraints of protein motion. ICoN generates conformations with low-energy, high-fidelity structural properties, enhancing the accuracy of conformational predictions beyond those captured by the original MD dataset (Ruzmetov et al., 2024).

### 4.3 Transformers (AphaFold pipelines)

Transformers excel by leveraging self-attention mechanisms that allow them to consider all residues in a sequence simultaneously (Wang and Li, 2024). This capability is essential for IDPs, where the interactions between distant residues—often characterized by high contact order—can significantly influence the overall conformation (Plaxco et al., 1998). The self-attention mechanism at the core of transformer models sets them apart from other DL techniques like CNNs and RNNs, by allowing it to dynamically weigh the influence of each residue in a sequence based on its transitory effect on every other residue, regardless of distance (Choi et al., 2023). This ability to capture long-range, non-linear interactions is particularly advantageous for IDPs, where such dynamic and non-local interactions are crucial for defining the protein’s conformational ensemble, leading to predictions that are both more comprehensive and reliable compared to traditional MD simulations or other DL models. Transformers can process entire protein sequences quickly and efficiently, generating a probabilistic distribution of possible conformations that reflects the inherent flexibility and diversity of IDPs (Ruff and Pappu, 2021). Moreover, transformers have a unique comparative advantage in their scalability and ability to learn from large, diverse datasets. Unlike RNNs, which may suffer from issues like vanishing gradients when dealing with long sequences, transformers maintain performance by processing sequences in parallel, making them highly scalable. AlphaFold (Jumper et al., 2021; Abramson et al., 2024) employs an advanced transformer architecture to capture complex sequence-structure dependencies, leveraging multi-head self-attention mechanisms to model the intricate spatial relationships among amino acid residues, thereby achieving unprecedented accuracy in predicting protein structure (Ruff and Pappu, 2021). Moreover, the advent of AlphaFold2 has fostered the development of various pipelines aimed at effectively modeling multiple conformational states or predicting conformational ensembles of both well-folded proteins and proteins exhibiting intrinsic disorder or intrinsic flexibility (Aranganathan et al., 2024; Fan et al., 2024; Ghafouri et al., 2024; Guan et al., 2024; Li et al., 2024). AlphaFold2 has demonstrated an emergent capability to capture alternative conformational states when guided by appropriate modifications to its multiple sequence alignment (MSA) inputs (del Alamo et al., 2022). Recent studies have demonstrated that by manipulating MSA depth and composition, AlphaFold2 can predict not just single structural states but entire ensembles of functionally relevant conformations, including those of IDPs and membrane proteins that undergo large-scale conformational shifts (Ghafouri et al., 2024). His suggests that AlphaFold2’s architecture inherently encodes information about sequence-driven conformational landscapes, albeit indirectly. Beyond standalone transformer-based architectures, recent advancements have explored hybrid approaches that combine transformers with other DL frameworks to improve IDP ensemble generation (Zhu et al., 2024a).

A transformer-based model developed by Chennakesavalu and Rotskoff, 2024 enhances the conformational sampling of IDPs by reconstructing atomic-resolution protein structures from backbone coordinates. The model integrates statistical side-chain conformations with a transformer architecture to generate realistic protein ensembles. Using a transformer that predicts side-chain configurations based on backbone dihedral angles, the model incorporates both local dihedral dependencies and global sequence-wide interactions. The model efficiently produces atomistic conformations consistent with MD simulations when applied to proteins like Chignolin and the IDR of the androgen receptor (AR-IDR) (Chennakesavalu and Rotskoff, 2024).

### 4.4 Diffusion models

Diffusion models represent another class of DL-based approachs that are being used for generating conformational ensembles of IDPs, as opposed to relatively older models like GANs (Ho et al., 2020). They leverage a probabilistic generative framework to learn the inherent structural diversity of IDPs with a higher fidelity when compared to previous frameworks. Diffusion probabilistic models operate through a two-step process involving forward diffusion and reverse denoising. In the forward process, noise is incrementally added to the data, transforming the protein conformations into a noisy latent representation. The reverse process then employs a neural network, typically inspired by architectures like transformers, to denoise the latent space iteratively, reconstructing plausible protein conformations (Zhang et al., 2024). This framework enables the generation of diverse ensembles directly from input sequences, without relying on multiple sequence alignments or extensive experimental data.

**FIGURE 2:**
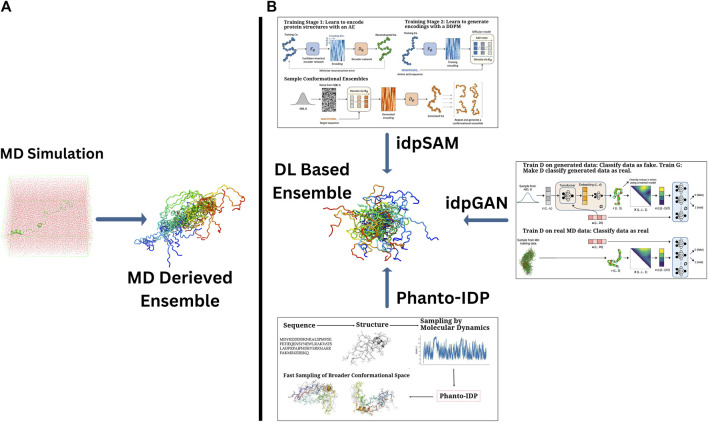
A comparative array of approaches of generating the conformational ensembles for IDPs. **(A)** Traditional MD simulations, which provide detailed conformational sampling of IDPs and **(B)** DL-based generative frameworks offering computationally efficient alternatives to derive IDP ensembles with comparable accuracy. Sub-images (boxes) in **(B)** portray the graphical abstracts of a few highlighted DL-based methods (taken directly from the corresponding papers with appropriate copyright permissions), namely, (i) idpGAN (GAN-driven ensemble generation from MD-based and learned distributions) (Janson et al., 2023), (ii) idpSAM (autoencoder-Diffusion-based sampling of conformations) (Janson and Feig, 2024), and (iii) Phanto-IDP sequence-to-ensemble modeling leveraging MD sampling for broader conformational exploration) (Zhu et al., 2024a).

The idpSAM model is a significant advancement, evolving from the earlier idpGAN architecture with transferability in mind. It integrates an AE and a denoising diffusion probabilistic model (DDPM) to enhance the generation of protein conformations. The AE compresses the 3D structural information of protein Cα coordinates into a lower-dimensional latent space. The DDPM then iteratively refines these noisy encodings, learning their probability distribution and improving the quality of generated conformations. After training, a decoder reconstructs 3D structures from these refined encodings, allowing idpSAM to produce highly accurate models of IDPs, including those not present in the training dataset (Janson and Feig, 2024). An overall display of these models and methods as compared to MD simulation is portrayed in Figure 2.

The IDPFold model utilizes a conditional diffusion model framework to generate protein conformational ensembles directly from their sequences. This generative framework consists of two primary processes: a forward diffusion process and a reverse denoising process. During the forward diffusion phase, Gaussian noise is progressively added to real protein structures in a series of small steps, effectively disrupting the native conformations and embedding them into a smooth latent space. This process mimics a stochastic trajectory that gradually removes structural details while retaining global sequence information. The reverse process, governed by a DL-based denoising network, reconstructs valid protein structures by iteratively refining the noisy representations back into physically meaningful conformations. IDPFold employs DenoisingIPA Blocks, inspired by AlphaFold2’s structural modules, to ensure that generated structures adhere to realistic backbone geometries and residue-residue interactions. Additionally, the model integrates sequence-based conditioning to guide the denoising trajectory toward ensembles that reflect the inherent flexibility and heterogeneity of IDPs. IDPFold employs a hybrid dataset comprising crystal structures, NMR structures, and MD trajectories for training and evaluation. IDPFold predicted ensembles achieve accuracy levels that are comparable to traditional MD simulations, and sometimes even higher while not being restricted by energy barriers during sampling (Zhu et al., 2024b).

Taneja and Lasker devised a two-stage generative pipeline. The first stage employs supervised models to predict ensemble-derived two-dimensional (2D) structural properties, such as pairwise distance maps and covariance matrices, by learning from sequences with closely related ensembles. These 2D features serve as low-dimensional representations of IDP conformations and guide the subsequent generative process. In the second stage, a diffusion model generates 3D CG conformational ensembles using learned 2D representations. The forward diffusion process gradually adds Gaussian noise to known conformations, transforming them into a latent distribution. The reverse diffusion process then removes this noise stepwise, reconstructing biologically plausible IDP ensembles. A neural network guides this denoising, ensuring structural realism while overcoming mode collapse, making diffusion models a robust alternative for IDP ensemble generation. This approach, trained on a dataset of CG MD simulations, demonstrated accurate 2D and 3D predictions closely mathcing experimental observables (Taneja and Lasker, 2024).

## 5 Overcoming the energy landscape in IDPs

Effectively sampling the conformational space in IDPs requires overcoming large energy barriers that separate diverse conformational states (Do et al., 2014). Traditional methods like REMD address this challenge by simulating multiple replicas of the system at different temperatures, allowing exchanges between replicas to enhance sampling (Qi et al., 2018). However, REMD is computationally expensive, limiting its applicability to large IDPs or long simulation timescales. Deep generative models offer a promising alternative, as they are not restricted by the topology of the potential energy landscapes and can explore conformational spaces more efficiently (Anstine and Isayev, 2023). BGs are a class of generative models specifically trained to sample configurations directly from the system’s energy function (Noé et al., 2019). Instead of explicitly learning the system’s probability density function (such as from short MD trajectories), these models are designed to sample configurations from an equilibrium distribution by leveraging a dimensionless energy function *u*(*x*). They achieve this through a generative network paired with reweighting procedures. The generative network transforms samples from a simple prior distribution *P*(*z*) (e.g., a Gaussian) in latent space into high-probability configurations from the target distribution *P*(*x*)∝*e* ^−*u*(*x*)^ (Zheng et al., 2023). One significant limitation of BGs is their tendency toward mode collapse, focusing on a limited number of low-energy metastable states and failing to explore the broader conformational landscape characteristic of IDPs (Patel and Tewari, 2022). Sparse training data and the complexity of IDP free energy landscapes exacerbate this issue, as these models tend to neglect transient or high-energy states (Noé et al., 2019). Therefore, energy-only learning biases sampling toward stable regions and lacks the diversity needed for IDP modeling. It has been suggested that training solely on energy functions may be insufficient to capture the extensive conformational diversity of IDPs (Patel and Tewari, 2022; Aranganathan et al., 2024). Recent advancements to BGs encompass equivariant flow matching models (Klein et al., 2023b) and transferable BGs (Klein and Noé, 2024), which establish a framework for incorporating molecular topology and symmetries into the energy function of the biomolecular system. But the key drawback remains that massive training datasets are required to explore essential modes without suffering from mode collapse (Aranganathan et al., 2024). Following the achievements of AF2, the Str2Str model employs a heating-annealing training technique for a score-matching model, facilitating navigation across energy landscape barriers (Lu et al., 2024). This method is exclusively trained on crystal structures and enables the simulation of local fluctuations similar to those observed in microsecond-long MD simulations. AlphaFlow/ESMFlow (Jing et al., 2024) leverages the AF2 network within a flow-matching framework and has been trained on both PDB and short MD datasets of 100 nanoseconds to incorporate timescale information into its training regimen to effectively capture local fluctuations (Aranganathan et al., 2024). The ConfDiff model integrates a force-guided diffusion framework and enhances the generation of diverse and high-fidelity protein structures. The model employs a regular forward diffusion - reverse diffusion setup where a DL network utilizes an additional force guidance mechanism to prioritize conformations with lower potential energy. This unique approach allows ConfDiff to align generated structures more closely with the Boltzmann distribution, effectively addressing the limitations of existing score-based diffusion methods that often fail to incorporate essential physical knowledge (Wang Y. et al., 2024).

Integrating energy-based constraints or regularization terms into other deep generative models has proven successful (Li et al., 2023). For example, idpGAN incorporates energy distributions from MD simulations during training (Janson et al., 2023). This acts as an implicitly learned energy constraint, guiding the model to generate ensembles with realistic energy profiles, thereby improving the accuracy of the generated conformational ensembles. IDPFold captures Boltzmann distributions, ensuring diverse sampling beyond metastable states (Zhu et al., 2024b). Its hybrid training approach—pre-training on experimental structures and fine-tuning on MD trajectories—enhances structural fidelity and flexibility, effectively avoiding energy barriers that limit BGs. By filtering non-physical structures and aligning free energy distributions with MD data, ICoN achieves thermodynamically consistent ensembles (Ruzmetov et al., 2024). Looking forward, the authors have expressed their interest in exploring energy-based training methods for Phanto-IDP to further enhance its performance (Zhu et al., 2024a).

## 6 Enhanced conformational sampling using AI in MD simulation

Conformational sampling using AI-enhanced MD simulations marks a transformative advancement in structural biology by synergizing the precision of physics-based models with the computational efficiency of ML and DL strategies (Zhang J. et al., 2023). Traditional MD simulations, governed by Newtonian mechanics, excel at providing atomistic insights into biomolecular dynamics but are intrinsically limited by their reliance on fine-grained time steps (Hollingsworth and Dror, 2018), which capture fast motions such as bond vibrations but fail to traverse biologically relevant timescales efficiently (Son et al., 2024). Enhanced sampling techniques, such as metadynamics, umbrella sampling, and replica exchange MD, aim to overcome these barriers by reweighting conformational distributions or sampling biased energy landscapes (Abrams and Bussi, 2014). However, these approaches often require detailed prior knowledge of the system, involve considerable computational expense, and are susceptible to missing critical transitions between metastable states. Integration of AI has contributed to the development of enhanced sampling techniques by addressing key limitations of the traditional methods (Prašnikar et al., 2024). For example, ML models trained to estimate free energy surfaces or bias potentials enable adaptive approaches to biased simulations, improving sampling efficiency (Galvelis and Sugita, 2017). RL algorithms have also been employed to optimize the initialization of sampling or the application of bias potentials (Shamsi et al., 2018; Zhang et al., 2018). Learned Replica Exchange (LREX) approach, where BGs are used to directly map high-temperature configurations to target temperatures, effectively bypassing the need for multiple intermediate replicas (Invernizzi et al., 2022). However, these approaches do not fix the problem of needing to re-run simulations from scratch for altered parameters (Aranganathan et al., 2024). Recent work by Brotzakis et al. (2025) exemplifies the power of AI-guided MD by integrating AlphaFold-predicted inter-residue distances as structural restraints within a Bayesian metainference framework. This approach, termed AlphaFold-Metainference (AF-MI), efficiently samples conformational ensembles of IDPs while maintaining agreement with SAXS and NMR data, overcoming the limitations of standalone AlphaFold predictions (Brotzakis et al., 2025).

Other than just AI-enhanced sampling procedures, other works have successfully integrated DL models into the MD simulation, such as DeepDriveMD (Lee et al., 2019), which is a deep convolutional variational autoencoder (CVAE) to cluster protein folding trajectories, all collated to a reasonably small number of conformational states. ITO (Implicit Transfer Operator) uses DDPMs to learn transition probabilities directly from MD data, enabling efficient simulation over larger time steps while preserving physical accuracy (Schreiner et al., 2023). Similarly, Timewarp is a normalizing flow-based generative model that learns to dynamically increase and optimize time steps upto a hundred femtoseconds at a time to accelerate the rate of MD when used for conformational sampling (Klein et al., 2023a). DiAMoNDBack employs a generative model to backmap CG protein structures to all-atom resolution. Using a diffusion-denoising process, it restores atomistic details while maintaining the integrity of the Cα trace to improve the resolution and accuracy of (Jones et al., 2023). Integrating various AI methods (Table 1) directly into the MD engine enjoys the advantage of being transferable and well generalized across most large, complex, and novel biomolecular test systems (Aranganathan et al., 2024).

**TABLE 1:** An overview of various AI-based methods for modelling IDP conformational ensembles, highlighting applicability, advantages, and limitations.

Method	Applicability in IDP sampling	Key advantages	Key limitations
*Recurrent Neural Networks (RNNs)*	Used for sequence-based structural predictions (including disorder). Can track sequential transitions in short IDRs or small peptides by updating hidden states over time. Used in DynamICE.	Suitable for time-series or short “fragment-based” conformational modeling. Straightforward to implement	Struggle with long IDP sequences (vanishing gradients). Less effective at capturing non-local residue interactions
*Variational Autoencoders (VAEs)*	Learn a latent space of conformations by compressing and reconstructing 3D structures for IDP ensembles. Used in ICoN	Provide a smooth, continuous latent representation, enabling easy interpolation between conformational states	Require large and high-quality training datasets. Transferability and generalizability are a concern
*Generative Adversarial Networks (GANs)*	Adversarial framework (generator vs. discriminator) to produce diverse structural conformations. Used in idpGAN.	Often yield high structural diversity in generated ensembles. Significantly fast at inference	Risk of mode collapse, where certain conformations dominate while others vanish
*Transformer-Based Architectures*	Sequence-to-structure models that leverage self-attention to handle long-range IDP interactions. Variants or pipelines can sample alternative conformations by altering input conditions or by adding constraints. Used in AF-Cluster/AF-MI.	Captures long-range dependencies effectively; helpful in modeling disordered regions	Often computationally heavy to train from scratch
*Diffusion Models*	Use a forward noise and reverse denoising process to sample 3D conformations from learned distributions. Used in IDPFold	Can systematically explore complex, high-dimensional spaces, often capturing rare states. Once trained, can generate large ensembles relatively quickly. Better diversity in predictions	Typically computationally complex to train (involving many forward–reverse steps). Must have sufficiently comprehensive training data to avoid generating unphysical conformations
*Graph Neural Networks (GNNs)*	Model proteins as graphs (residues = nodes, edges = interactions). Useful for capturing local and nonlocal residue-residue interactions in flexible/disordered domains. Used in Phanto-IDP.	Naturally incorporate 3D connectivity. Good at capturing subtle structural relationships in IDP ensembles (e.g., contact maps, side-chain interactions)	Still relatively new for IDP ensemble generation; typically combined with other generative frameworks. Requires abundant structural data for robust training
*Reinforcement Learning (RL)*	Optimizing IDP folding pathways and ensemble sampling. DynamICE uses a form of RL to integrate experimental data for inference	Can learn optimal pathways for conformational switching	Requires extensive training and well defined reward functions
*Flow-Based (Normalizing Flows & Boltzmann Generators)*	Invertible transformations from a simple prior (e.g., Gaussian) to a complex IDP distribution. BGs integrate energy functions to sample near Boltzmann equilibrium. Used in AlphaFlow/ESMFlow	Can capture rare states missed by short MD. Exact density estimates enable reweighting or free-energy analysis	Computationally intensive for large IDPs. Mode collapse or incomplete sampling if data or hyperparameters are insufficient. Requires accurate energy functions for BGs

## 7 Comparative efficiency: DL versus MD

While MD simulations provide detailed, physics-based insights into protein dynamics, they are notoriously resource-intensive. Simulating a single IDP to capture its complete conformational landscape can require continuous operation on high-performance computing (HPC) clusters for weeks or even months (Shaw et al., 2008; Hollingsworth and Dror, 2018). Studies have shown that adequately exploring the conformational space in IDPs via MD often demands several thousands of CPU hours. Despite the extensive computational resources involved, MD may still fail to capture rare but biologically significant conformational states, which are crucial for understanding the functional roles of IDPs in processes such as protein-protein interactions and the formation of transient complexes (Gopal et al., 2021). In contrast, DL models offer a scalable and far more time-efficient alternative, which are particularly evident in high-throughput analyses. After an initial training phase—which might require substantial computational power (hundreds of GPUs) over a period of many days (Cheng et al., 2023), particularly when processing large datasets like those from the PDB—DL models can predict IDP conformational states in seconds or minutes (Gupta et al., 2022). For instance, a recent study demonstrated that a DL model, trained on IDP conformations, could generate accurate ensemble of 300 conformations in under 20 min, a process that could take several days to achieve through MD simulations (Zhu et al., 2024b). The front-loaded computational cost of training DL models is offset by the remarkable speed of the inference phase, which can be executed on less powerful hardware, such as a standard GPU, significantly reducing ongoing computational demands (Alzubaidi et al., 2021).

**FIGURE 3:**
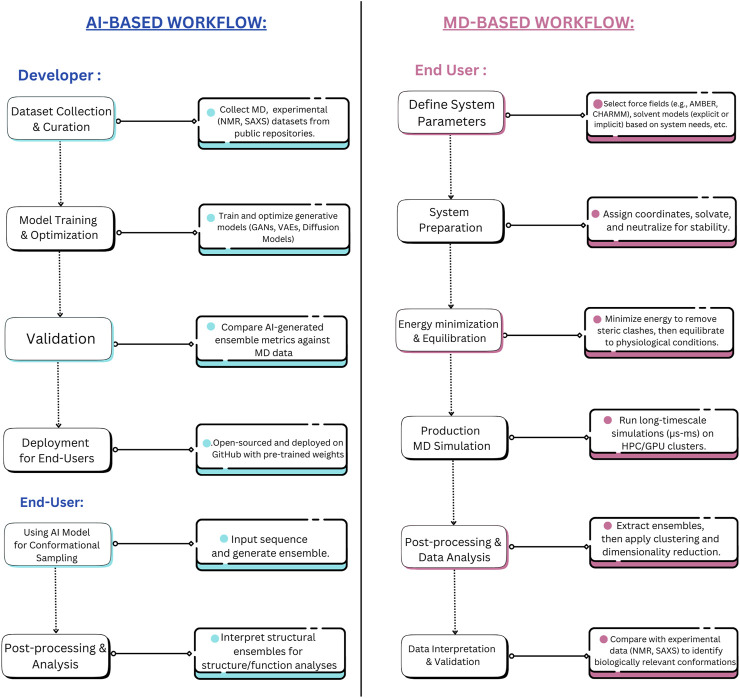
Developer and End-user level tasks for AI-Based Methods vs. MD Simulations for IDP Conformational Sampling. This figure contrasts the workflow differences between AI-based methods (transferable e.g., idpGAN, idpSAM, ICoN, IDPConformerGenerator, etc.) and MD simulations, distinguishing the roles of developers and end-users in each approach. AI-based methods streamline conformational sampling by shifting the complexity to the development phase while allowing rapid inference at deployment. Whereas, MD simulations necessitate a lengthier (and time-intensive) process. The developer-side complexity of MD simulations, including force field optimization, solvent modeling, MD engine (GROMACS, LAMPPS, etc.) parameterization, etc., is not fully depicted to maintain clarity and avoid visual congestion.

Beyond their computational efficiency, DL models excel in adaptability and continuous improvement. They can be updated with new data as it becomes available, enhancing their accuracy without the need to rerun simulations from scratch (Taye, 2023). This is particularly advantageous when integrating new experimental data from cryo-EM, or NMR spectroscopy, or from curated IDP databases (Evans et al., 2023; Giri et al., 2023). Updating DL models involves fine-tuning the model parameters using optimization algorithms on new training data, allowing the model to adapt to changes without complete retraining (Prapas et al., 2021). This process typically utilizes transfer learning techniques, where pre-trained weights are adjusted based on new datasets, significantly enhancing prediction accuracy while maintaining computational efficiency (Koval et al., 2023). Such integration refines the model’s predictions and further enhances its utility, a capability that contrasts sharply with MD simulations, which typically require starting anew for each modification or new experimental condition. This adaptability makes DL models highly suitable for dynamic research environments where conditions and data are constantly evolving (Nikolados et al., 2022). The outputs from DL-based IDP conformational sampling tools typically include a set of predicted conformations along with their associated probability distributions, energy scores, and structural metrics to evaluate the relative stability and likelihood of different conformers (Teixeira et al., 2022; Brown et al., 2024). Figure 3 portrays the comparative workflows of MD vs. AI approach towards sampling of conformational spaces in IDPs from an end-user and a developer perspective.

The recent development of Phanto-IDP offers compelling qualitative and quantitative evidence in favour of AI-based methods. In a systematic benchmarking provided by Zhu et al. (2024c), Phanto-IDP demonstrated superior performance across multiple metrics compared to both MD and prior DL models (Zhu et al., 2024a). For instance, on the IDP system α-synuclein (140 residues), Phanto-IDP achieved an average backbone reconstruction RMSD of 2.714 Å, significantly lower than the authors’ older VAE-based (Zhu et al., 2023) models’ 10.417 Å, when compared to the reference ensemble sampled by MD (see Table 1 of Zhu et al., 2024a). Furthermore, the Jensen-Shannon (JS) divergence of the Rg distributions of Phanto-IDP-generated ensembles when compared with MD-derived ensembles was 0.105, indicating excellent alignment with Boltzmann-distributed conformational diversity, much better than that of their previous model showing a divergence of 1.223. Critically, Phanto-IDP generated 50,000 conformations in under 50 s on a single GPU, i.e., much faster than the 270 h required for a 1 µs MD simulation even if *ab initio* training is taken into consideration—while still preserving global properties like ensemble diversity and Rg distributions after side-chain refinement (see Figure 4 of Zhu et al, 2024a). Enhanced sampling capabilities were clearly demonstrated by Phanto-IDP through its ability to identify rare conformational states. When trained on short MD trajectories, the model generated approximately 5.6% helicity-rich conformations that were entirely absent from the training data—approaching the 18.3% observed in REMD—even though it omits the iterative, physics-based sampling typically required to overcome kinetic barriers (see Figure 6 of Zhu et al, 2024a). Moreover, PCA analysis revealed that the conformational space covered by Phanto-IDP is considerably broader, including regions unvisited by traditional MD (see Figure 4 of Zhu et al, 2024a), which confirms the enhanced diversity of the generated ensembles. This broader sampling not only underscores Phanto-IDP’s capacity to traverse high-energy barriers but also its potential to capture biologically relevant states that conventional MD might miss. While J-coupling values for Phanto-IDP ensembles aligned better with experiments in systems like RS1 and drkN SH3, MD outperformed the model in others (see Supplementary Figure S16 of Zhu et al, 2024a), highlighting context-dependent accuracy. Even though more training and fine-tuning is required as acknowledged by the authors, it shows AI-methods’ promise in achieving near-experimental accuracy.

The method idpGAN demonstrated rapid sampling speeds where it could generate thousands of conformations within 1 s of wall-clock time for IDPs lower than 150 residues as shown in the authors’ Supplementary Figure 25 (Janson et al., 2023). This speed advantage makes idpGAN orders of magnitude faster than MD; however, it struggled with ensemble fidelity, particularly in recovering MD ensemble distributions and Rg statistics, leading to inconsistent transferability. To address these shortcomings, the idpSAM was developed as a successor, leveraging a diffusion-based framework that improved ensemble fidelity while maintaining high computational efficiency (Janson and Feig, 2024). IdpSAM achieved an optimal trade-off between speed and accuracy at 100 diffusion steps, generating 10,000 conformations in ∼4 min while still far outperforming the total 509 CPU hours required for 5 MCMC-based IDP sampling. IDPFold exhibited the best experimental agreement among these approaches (Zhu et al., 2024b). While idpSAM was faster (0.63 min for 300 conformations) as evident in their Table (see Table 1 of Zhu et al., 2024b), its structures were overly compact and deviated from experimental values in terms of Rg for certain IDP systems. In contrast, IDPFold achieved a lower mean absolute error (MAE) of 0.48 compared to MD’s 0.59 in chemical shift predictions, indicating superior accuracy. Furthermore, IDPFold extended sampling beyond metastable MD states, capturing a broader Boltzmann-distributed ensemble to an extent (see Figure 3 of Zhu et al, 2024b), making it more representative of experimentally observed IDP behavior. While idpSAM faithfully reproduced its MD-derived training ensembles, IDPFold provided a more experimentally accurate and diverse conformational landscape, albeit at a higher computational cost (∼21 min). A look-up summary Table (Supplementary Table S1) of comparison pertaining the above discussed results is added in the Supplementary Materials.

## 8 Disadvantages of DL over MD simulations

Despite the numerous advantages of DL models in sampling conformational ensembles of IDPs, they also present several notable disadvantages compared to traditional MD simulations. Firstly, DL models are heavily dependent on the quality and diversity of the training data (Munappy et al., 2022). Inadequate or biased datasets can lead to models that fail to generalize well to novel or underrepresented IDP sequences, potentially missing critical conformational states. Secondly, the interpretability of DL models remains a significant challenge (Liu and He, 2024). Unlike MD simulations, which are grounded in physical principles and provide explicit insights into atomic interactions, DL models often operate as “black boxes,” making it difficult to understand the underlying mechanisms driving their predictions (Samek et al., 2019). Thirdly, DL models require substantial computational resources and expertise for their development and training. Constructing and fine-tuning these models necessitates advanced knowledge in ML and access to powerful hardware, which may not be readily available to all research groups (Sarker, 2021). Additionally, DL models are susceptible to overfitting, especially when trained on limited datasets. Overfitting can result in models that perform exceptionally well on training data but poorly on unseen data, undermining their reliability for predictive applications (López et al., 2022). Lastly, the physical accuracy of DL-generated conformations can sometimes be compromised, as these models may prioritize statistical patterns over thermodynamic plausibility, leading to predictions that, while statistically likely, may not always reflect biologically relevant states (Wodak et al., 2023). While DL models offer several advantages in generating conformational ensembles of IDPs, including their computational efficiency and ability to predict a broad array of conformations from sequence data alone, it is important to recognize that these models cannot completely replace MD simulations (Gomes et al., 2020; Lindorff-Larsen and Kragelund, 2021). DL-based approaches often rely on training datasets derived from MD-generated conformational ensembles, and their accuracy is intrinsically linked to the quality and diversity of the data they are trained on (Zheng et al., 2023). Without continuous updates and supplementation with new experimental or simulated data, DL models risk generating outdated or biased predictions (Gichoya et al., 2023), particularly as they struggle to generalize well to novel protein sequences or biological conditions not represented in the training data (Janson et al., 2023; Janson and Feig, 2024; Ruzmetov et al., 2024). A table of comparison is given (Table 2) to illustrate the pros and cons of AI-based methods and MD simulations for IDP conformational sampling.

**TABLE 2:** Pros and cons of AI-Based methods and MD simulations for IDP conformational sampling.

Feature	AI-BASED methods	MD simulations
*Computational Efficiency*	Pros: Generates large ensembles rapidly (minutes to hours) once the model is trained	Cons: Simulation of medium-to-large systems is computationally expensive and often requires HPC clusters
*Sampling Capability*	Pros: Efficiently explores vast conformational spaces, including rare and transient states Cons: Sampling quality is heavily dependent on the size and quality of the training dataset.	Pros: Ensures thermodynamic accuracy in sampled states Cons: Rare state sampling is limited by inherent timescale constraints and energy barriers, making it challenging to capture less populated conformations
*Physical Accuracy*	Cons: Relies on statistical approximations that may not fully capture physical laws unless physics-based learning or constraints are incorporated into the predictive model	Pros: Based on physics-driven force fields, providing consistent and realistic atomistic behaviour when paired with the proper choice of water model and force field
*Scalability*	Pros: Scales efficiently with large datasets and supports rapid inference	Cons: Despite parallelization, the computational cost increases steeply with system size and simulation duration
*Data Dependence*	Cons: Requires extensive, high-quality training data; performance can degrade with biased or limited data	Pros: Operates independently of external training data, relying solely on physical models
*Experimental Validation*	Pros: Can integrate experimental constraints (e.g., NMR, SAXS, etc.) to guide or refine predictions Cons: Even with integrated constraints, the models typically require independent experimental validation to ensure that predictions are accurate and not overly biased by the training data	Pros: Simulation outcomes can be directly compared with experimental observables for validation Cons: Despite high accuracy, limited simulation timescales can make it challenging to capture rapid or rare dynamic events, potentially leaving out key aspects of the conformational ensemble
*Flexibility*	Pros: Easily adapted to various IDP types using techniques like transfer learning	Cons: Requires re-simulation for different conditions or system modifications
*Interpretability*	Cons: Often perceived as a “black box,” which can complicate mechanistic understanding	Pros: Provides clear mechanistic insights into atomic interactions and dynamics, facilitating interpretation of the molecular behaviour

## 9 Applications and case studies: deep learning in IDP research

One significant advancement in the field of DL-based conformational ensemble generation for IDPs is the recent inclusion of ensembles generated by methods such as idpGAN and IDPConformerGenerator (Teixeira et al., 2022) in the PED (Ghafouri et al., 2024). This marks a pivotal shift from the previous focus solely on ensembles derived from explicit experimental data, such as those obtained through MD simulations. These methods are particularly useful for disordered proteins like Amyloid-beta (Aβ) (Scollo and Rosa, 2020) and α-synuclein (Williams et al., 2018), which play key roles in neurodegenerative diseases such as Alzheimer’s and Parkinson’s disease respectively. Aβ is a disordered peptide involved in Alzheimer’s disease, and its structural ensemble has been extensively studied (Balupuri et al., 2020).

Brotzakis et al. (2025) introduced a significant advancement by integrating AlphaFold-derived inter-residue distances as structural restraints within a Bayesian inference framework (Metainference) (Brotzakis et al., 2025). This method refines MD simulations by leveraging DL-based structural constraints, resulting in ensembles that exhibit greater agreement with experimental data compared to conventional MD approaches alone. A key application of this method was demonstrated in the structural characterization of both full disordered proteins (e.g., Aβ, α-synuclein, etc.) and partially disordered proteins (e.g., TDP-43, ataxin-3, human prion protein), where AlphaFold-predicted distance distributions were employed as guiding restraints in Metainference-enhanced MD simulations. Quantitative benchmarking against experimental data demonstrated that AF-MI ensembles substantially reduced deviations in inter-residue distance distributions (e.g., KL divergence of 0.018 for TDP-43 vs. 0.582 for standalone AlphaFold) and improved agreement with SAXS-derived radius of gyration (Rg) values (Supplementary Figure S4 of Brotzakis et al., 2025), confirming the physical relevance of AlphaFold-derived restraints. Furthermore, computational efficiency was markedly improved, as AF-MI simulation converged significantly faster than conventional MD. Beyond improving accuracy and efficiency, the ability to sample a broader conformational ensemble was significantly enhanced, as the Metainference-assisted ensembles exhibited significant increase in conformational heterogeneity compared to MD-only simulations. This is evident from the pairwise distance distributions in Figure 2 of Brotzakis et al., 2025, where AF-MI better reproduces SAXS-derived experimental distributions compared to conventional MD and CALVADOS-2 (Lindorff-Larsen and Kragelund, 2021), a CG IDP simulation model. Additionally, SAXS validation across multiple proteins (Figures 3–6 of Brotzakis et al., 2025) demonstrates that AF-MI yields ensembles that more accurately capture experimentally observed disorder, supporting its ability to explore diverse conformational states. AF-MI was also compared against CALVADOS-2 through a direct experimental validation via SAXS. For four of six partially disordered proteins (e.g., ataxin-3, KL = 0.020 vs. 0.042 for CALVADOS-2), AF-MI ensembles aligned more closely with SAXS data, while matching CALVADOS-2’s performance in the remaining two cases (e.g., TDP-43, KL = 0.018). Notably, this approach corrected structural inaccuracies in standalone AlphaFold predictions (e.g., overly compact conformations in disordered regions), resulting in ensembles that better reproduced NMR chemical shifts and SAXS-derived scaling exponents. These findings suggest that AF-MI is not only computationally efficient (compared to only MD) but also capable of generating more biologically relevant IDP ensembles than existing CG models.

Amyloid-β1-42 (Aβ42) aggregation is a key pathological hallmark of Alzheimer’s disease, with oligomerization and fibril formation being driven by specific structural rearrangements. Among these, salt-bridge interactions, particularly D23–K28, are hypothesized (Ruzmetov et al., 2024) to regulate aggregation propensity. Experimental studies have suggested that this interaction promotes β-sheet stabilization and enhances fibril formation, but due to the transient and heterogeneous nature of Aβ42 monomers, its precise conformational landscape has remained elusive. Ruzmetov et al. (2024) introduced ICoN, a DL-based generative model, which efficiently sampled the wide conformational space of Aβ42 beyond what was observed in MD simulations. ICoN uncovered previously unseen conformations that featured the D23–K28 salt-bridge—a structural arrangement that was rarely observed in the MD trajectory despite extensive sampling efforts, revealing a spectrum of previously inaccessible conformational substates stabilized by the D23–K28 interaction. This discovery validated experimental hypotheses about the role of D23–K28 in aggregation but provided a new level of structural resolution by demonstrating multiple viable conformational states, ranging from extended to highly compact monomeric arrangements, which may serve as precursors for fibril formation. Additionally, ICoN revealed key structural motifs in Aβ42 monomers, including four local bends at residue positions 4–6, 11–14, 26–28, and 36–38. While these turns have been individually reported in prior studies, their simultaneous presence in the AI-generated conformations suggests a potential role as structural precursors for fibril assembly, thus necessitating further studies. Furthermore, ICoN identified an alternative E22–K28 salt-bridge conformation, which is hypothesized to suppress the toxic β-hairpin formation at residues E22–D23 and reduce aggregation propensity (Nasica-Labouze et al., 2015). This AI-generated ensemble provided structural models that could not be derived solely from MD, offering novel mechanistic insights into Aβ42 aggregation dynamics. The ability to sample such rare states computationally is critical for designing aggregation inhibitors and therapeutics targeting early-stage oligomer formation.

In another study focused on three IDPs—polyglutamine Q15, Amyloid-beta 40 (Aβ40), and ChiZ from *Mycobacterium* tuberculosis—researchers employed DL-based AEs to generate conformational ensembles (Gupta et al., 2022). The AEs were trained on a limited dataset from short MD simulations, minimizing training time while maintaining the quality of the resulting conformations. The AEs demonstrated a marked ability to generate full conformational ensembles that accurately reproduced the experimental data and covered all conformations sampled in long MD simulations. For Q15 and Aβ40, the multivariate Gaussian model applied in the latent space enabled high-quality conformational reconstructions, with RMSD of around 5 Å and 6 Å, respectively. The generated ensembles effectively captured the diversity of the MD-sampled conformations, particularly in the smaller IDPs like Q15. Despite the challenges presented by larger proteins like ChiZ, where reconstruction RMSDs were higher (∼7 Å), the generative AE approach still outperformed traditional MD simulations by rapidly expanding the conformational space without extensive computational overhead. The results were validated through SAXS profiles and NMR chemical shifts, further highlighting the potential of DL in mining the conformational landscapes of complex IDPs.

## 10 Discussion and future directions

Recent breakthroughs in AI has shifted the *status quo* in protein structure and function prediction. The 50 year old problem of predicting proteins’ complex structures has largely been addressed by the likes of AlphaFold, RoseTTA Fold and others and the field has been awarded a part of the Nobel Prize in Chemistry 2024. However, most of the work in this field has largely been on well defined structured proteins and IDPs remain largely unexplored (Trivedi and Nagarajaram, 2022). Studying conformational ensembles of IDPs remain crucial to understanding their intrinsic flexibility allowing them to engage in a variety of biological functions such as signalling and molecular recognition, which are often mediated transient interactions with other biomolecules (Krieger et al., 2014). Additionally, aberrant behavior of IDPs is often linked to various diseases, including neurodegenerative disorders and cancers (Martinelli et al., 2019). Understanding the conformational dynamics of IDPs can help elucidate the molecular basis of these diseases and identify potential therapeutic targets (Abyzov et al., 2022). Traditional MD simulations and their various modifications have been extensively used for sampling the conformational ensembles of IDPs, however their shortcomings stand bold and clear (Zhu et al., 2023; Janson and Feig, 2024). Use of AI based tools in generating or sampling the various conformational states of IDPs has emerged as a key new frontier with distinguished advantages (Zhu et al., 2024b). For this, AI methods have been integrated in enhanced sampling techniques from MD simulations, as well as, integrated directly into the MD engines (Aranganathan et al., 2024; Prašnikar et al., 2024). Much effort has been dedicated to generative models such as GANs, VAEs, diffusion models, and others (Ruzmetov et al., 2024). Many methods have also been devised to sample IDP conformational states using AlphaFold pipelines (Ghafouri et al., 2024). Sampling most of the possible biologically relevant conformations of IDPs just from their protein sequence as an input is the ultimate goal but a gargantuan task. But already we have seen steady progress in this endeavour (Zheng et al., 2023). A common objective of most generative models, employed for the generation of the conformational states of IDPs, is to learn a low-dimensional latent representation of the high-dimensional conformational space of proteins to efficiently generate realistic and diverse conformational ensembles (Zhu et al., 2024a; Janson and Feig, 2024; Zhu et al., 2024c). Incorporating physics-based calculations into generative DL models is increasingly recognized as essential for developing approaches that yield predictions with higher accuracy and biological relevance (Raissi et al., 2019; Jagtap et al., 2020; Yang et al., 2020; 2022).

Although DL models can efficiently predict protein conformations and improve the speed of conformational ensemble generation, they remain limited by their reliance on pre-existing datasets (Vignesh et al., 2024). As a result, while AI-based methods are a powerful tool for exploring protein conformational landscapes, they should be considered complementary to, rather than a replacement for, traditional MD simulations. AI-based conformational studies of IDPs, such as α-synuclein, Tau protein, and amyloid-β, hold significant promise for elucidating the molecular basis and pathophysiology of diseases like Alzheimer’s and Parkinson’s (Sengupta and Kayed, 2022; Brotzakis et al., 2023). These studies can also aid in modelling novel and targeted therapeutic approaches, enhancing drug discovery efforts (Joshi and Vendruscolo, 2015). Future efforts should focus on integrating thermodynamic constraints directly into generative models to improve the accuracy and biological relevance of the generated conformations, since it has already be shown that learning the energy function along is not enough (Zheng et al., 2023). Most of the models today are capable of accurately sampling only relatively smaller IDP sequences. Larger IDP (including IDRs in large proteins) sequences can form non-trivial local structures which show transient long-range interactions within its sequence which are essential in understanding the underlying phenomena (Wohl and Zheng, 2023). Future research should explore scaling generative models to larger IDPs, (also pertaining to the IDRs present in large proteins and their intramolecular interactions) potentially by using hierarchical approaches that break down long sequences into smaller segments.

Most of the DL based tools made to predict the conformational ensembles of IDPs rely on training on simulated data i.e., CG or all-atom MD simulations and then validation via experimental data (Janson et al., 2023). While this paradigm has shown significant progress and promise, the other avenue i.e., training both on simulated data and experimental observables have been relatively less explored (Liu et al., 2024b). To ensure the reliability of AI-predicted conformational ensembles, rigorous experimental validation is essential. NMR ensemble fitting methods, such as back-calculated chemical shifts, J-couplings, and nuclear Overhauser effects (NOEs), allow direct comparison of predicted and experimentally observed data (Nerli et al., 2018). In addition, SAXS profiles serve as a complementary validation approach, offering global structural insights that can assess the compactness and overall conformational heterogeneity of predicted ensembles (Chinnam et al., 2023). Single-molecule Förster resonance energy transfer (smFRET) experiments further enable validation by capturing long-range distance constraints within IDPs, making them particularly useful for assessing the dynamic fluctuations of predicted structures (Qiao et al., 2021). These experimental techniques, when integrated with AI models, facilitate an iterative refinement process where structural predictions are continuously updated to maximize agreement with physical measurements (Qin et al., 2024). Recent efforts have focused on incorporating these experimental constraints directly into AI pipelines, ensuring that generative models not only sample plausible conformations but also converge toward physically meaningful ensembles that adhere to Boltzmann-weighted distributions (Liu et al., 2024a). Future advancements in AI-driven IDP modeling should prioritize the direct incorporation of experimental validation as an intrinsic component of model training and optimization, ultimately leading to more accurate and experimentally consistent ensemble predictions. DynamICE is an AI based tool developed that learns the probability of succeeding residue torsions from the preceding residue of the input sequence by employing a generative recurrent neural network (GRNN) model to build new conformational states of an IDP ensemble (Zhang O. et al., 2023). DynamICE (dynamic IDP creator with experimental restraints) distinguishes itself by taking advantage of experimental data types such as three-bond J-couplings, NOEs, and paramagnetic resonance enhancements (PREs) from NMR spectroscopy to bias the probability distributions of torsions of the GRNN (Lincoff et al., 2020). It evolves the structural ensembles dynamically by refining conformations through reward-based feedback, ensuring consistency with experimental data, rather than reweighting pre-existing static pools. A recent advancement by the same group, IDPForge (Intrinsically Disordered Protein, Folded and disordered Region Generator), leverages a transformer-based diffusion framework to generate all-atom conformational ensembles of IDPs and IDRs while preserving folded domains. Unlike DynamICE, which relies on torsional representations, IDPForge operates in Cartesian space, enabling direct integration of distance-based experimental restraints (e.g., PREs, NOEs) during generation. It eliminates sequence-specific training and achieves competitive agreement with NMR, smFRET, and Rg data while sampling transient secondary structures and rare conformations (Zhang et al., 2024). ExEnDiff is a model that employs an experiment-guided diffusion framework, where a stochastic differential equation is utilized to perturb protein data distributions towards a Gaussian distribution. By integrating experimental measurements from techniques such as NMR and SAXS, ExEnDiff corrects the sampling process to ensure that generated conformations align with physical realities and the Boltzmann distribution (Liu et al., 2024b).

Despite these advances in experiment-driven AI methods, the dynamic, loosely defined binding interfaces of IDPs continue to pose challenges for classical structure-based druggability metrics. Unlike folded proteins, IDPs (fuzzy) typically lack the stable pockets or well-formed hydrophobic clefts essential for conventional computational screens (Saurabh et al., 2023). Nonetheless, above developments highlight that if experimental observables can be incorporated to capture disordered-state ensembles, then transient druggability features can also be systematically modeled. The ability of AI tools to evolve structural ensembles using experimental restraints implies that subtle binding hot spots, allosteric regulatory sites, and disorder-to-order transitions may be identified and assessed for therapeutic potential. Expanding these pipelines to recognize ephemeral pockets, ligand-induced conformational shifts, and other IDP-specific druggability signatures could substantially enhance the predictive accuracy of AI-based frameworks (Lindorff-Larsen and Kragelund, 2021). Future efforts should explore to incorporate experimental constraints directly into DL pipelines to gradually evolve the structural ensemble prediction based on both simulated data and experimental observables. Further comparative studies on biological accuracy, thermodynamic relevance, and performance across the two broad paradigms will be crucial in determining whether a balanced reliance on both experimental and simulated data is most effective, or if prioritizing one data type over the other is more beneficial for generating accurate IDP conformational ensembles. Apart from iterative improvement of existing AI based models and using newer learning methods, it is hard to foresee how the generative ML models of predicting conformational ensembles of IDPs will evolve, or how generally applicable these models will be to the full range of protein behaviours critical to biological processes. Additionally, the transferability of generative models to novel sequences or different environmental conditions remains an open question. Even though this field of research is relatively new, there is no doubt that the further development of AI tools and their subsequent application will revolutionise the conformational sampling of IDPs both by enhancing MD simulation strategies and conformational ensemble prediction by generative methods (Ruzmetov et al., 2024).

## Footnotes

1 In the context of this review, IDPs refer to both completely and partially disordered proteins (IDPs, IDPRs).
