# Supplementary material for: Use of AI-methods over MD simulations in the sampling of conformational ensembles in IDPs
Source: Front Mol Biosci. 2025 Apr 8;12:1542267. doi: 10.3389/fmolb.2025.1542267 (PMC12011600; doi:10.3389/fmolb.2025.1542267)
Supplement: Supplementary file 1 [file DataSheet1.pdf]

Supplementary Materials for

Use of AI-Methods over MD Simulations in the Sampling of Conformational Ensembles in IDPs

by

Souradeep Sil, Ishita Datta, Sankar Basu*

Paper & Reference	Accuracy	Computational Efficiency	Ensemble Diversity	Rare State Sampling	Experimental Agreement
idpGAN<br>(Janson et al., 2023)	- Recovers contact maps $\log(p_{i,j})$ with low MSE_c across several IDPs (e.g. 0.057 for his5, 2.724 for protac).<br>- Captures sequence specific Rg distributions that closely track MD.	- Thousands of conformations in ~1 second (GPU), extremely fast.<br>- Minimal overhead after training.	- Broad coverage in PCA space for tested IDPs.<br>- Generally high variety, but the authors note some subtle states may be under represented with limited training data.	- Captures typical states but sometimes misses subtle or rare conformations (mode collapse).	- No direct experimental validation.<br>- Primary validation vs. coarse grained or ABSINTH MD references.<br>- Shows strong agreement on contact maps, Rg distributions and free energy landscapes relative to MD.
Phanto-IDP<br>(Zhu et al., 2024a)	- For smaller IDPs: ~1 Å backbone RMSD reconstruction, e.g. 0.885 Å on PaaA2 vs. MD reference.<br>- For α-syn, ~2.7 Å average RMSD vs. MD reference.	- 50,000 conformations in <1 min on a single GPU once trained, extremely fast inference.	- PCA coverage rivals that of MD.<br>- Cluster centroids vary widely.	- Generates conformations missing in MD (e.g., saddle points).	- Verified vs. NMR (dihedral angles, chemical shifts) & Rg; tends to match experiment well for tested IDPs.<br>- J-coupling errors in 4/10 systems.
idpSAM<br>(Janson and Feig, 2024)	- MSE_c = 3.30 vs. idpGAN's 4.52 MD's 7.79 (same number of training IDRs for both AI methods).<br>- Captures mutant vs. wild-type sequence differences.	- 10,000 conformations in ~4 min on a GPU with 100 diffusion steps. Slower than idpGAN.<br>- Higher transferability than predecessor.	- Matches MCMC contact maps and α-torsion distributions.	- Fails for helicity-rich ak37 unless trained on similar sequences.	- Validated against CG MD ensembles<br>– Rg underestimation inherited from ABSINTH references.
IDPFold<br>(Zhu et al., 2024b)	- MAE in chemical shifts ~0.48 vs. MD's 0.59 (better match).<br>- Showed consistently high ensemble validity amongst others.	- ~21 min to generate 300 conformations on one GPU. Slower but often more accurate than idpSAM/idpGAN.	- PCA overlaps with MD/REMD.<br>- Wider φ–ψ sampling than MD.<br>- Captures broad Boltzmann-like distributions. improving on metastable states.	- Generates transitions (e.g., structured ↔ disordered) not in short MD.	- Closer to experiment on Rg and NMR shifts than other one-shot.<br>- Some systems show near-experimental accuracy.

Table S1. Comparison of recent deep generative models for producing IDP ensembles. *Each row references a specific method described in its publication, while each column highlights key metrics: Accuracy, Computational Efficiency, Ensemble Diversity, Rare State Sampling and Experimental Agreement.*

References

Janson, G., and Feig, M. (2024). Transferable deep generative modeling of intrinsically disordered protein conformations. *PLOS Computational Biology* 20, e1012144. doi: 10.1371/journal.pcbi.1012144

Janson, G., Valdes-Garcia, G., Heo, L., and Feig, M. (2023). Direct generation of protein conformational ensembles via machine learning. *Nat Commun* 14, 774. doi: 10.1038/s41467-023-36443-x

Zhu, J., Li, Z., Tong, H., Lu, Z., Zhang, N., Wei, T., et al. (2024a). Phanto-IDP: compact model for precise intrinsically disordered protein backbone generation and enhanced sampling. *Briefings in Bioinformatics* 25, bbad429. doi: 10.1093/bib/bbad429

Zhu, J., Li, Z., Zhang, B., Zheng, Z., Zhong, B., Bai, J., et al. (2024b). Precise Generation of Conformational Ensembles for Intrinsically Disordered Proteins Using Fine-tuned Diffusion Models. 2024.05.05.592611. doi: 10.1101/2024.05.05.592611
